# Prevalence, correlates, and predictive value of high-risk human papillomavirus mRNA detection in a community-based cervical cancer screening program in western Uganda

**DOI:** 10.1186/s13027-019-0230-0

**Published:** 2019-05-14

**Authors:** Miriam Nakalembe, Philippa Makanga, Frank Mubiru, Megan Swanson, Jeffrey Martin, Megan Huchko

**Affiliations:** 1 Department of Obstetrics and Gynecology, Infectious Diseases Institute, Makerere University Kampala, Kampala, Uganda; 2 Department of Obstetrics and Gynecology, University of California, San Francisco, Uganda; 3 Department of Epidemiology and Biostatistics, University of California, San Francisco, Uganda; 4 Department of Obstetrics and Gynecology, Global Health Institute, Duke University, Durham, North Carolina USA

**Keywords:** Cervical cancer, Community-based screening, Human papillomavirus, mRNA testing, Predictive value, Uganda, Africa

## Abstract

**Background:**

New strategies are needed to combat the high incidence of cervical cancer in resource-limited settings such as sub-Saharan Africa. Screening for high-risk human papillomavirus (hrHPV) DNA is sensitive for pre-cancer, but its lack of specificity results in substantial overtreatment in low resource settings where additional testing (e.g., colposcopy) is rarely available. Testing for hrHPV E6/E7 mRNA may enhance specificity, but little is known about its performance characteristics in resource-limited settings.

**Methods:**

In a series of community health fairs in rural Uganda, women aged 25 to 49 years provided self-collected vaginal samples, which were tested for hrHPV (types 16, 18, 31, 33, 35, 39, 45, 51, 52, 56, 58, 59, 66 and 68) E6/E7 mRNA with the Aptima® assay. Positive specimens underwent testing for HPV-16 and 18/45. After excluding pregnant women, all women testing positive for any hrHPV subsequently were offered cervical biopsy to determine pathology.

**Results:**

A total of 1892 women provided a vaginal sample for hrHPV testing during 24 health fairs. The median age was 34 years, HIV prevalence was 10, and 95% had not been previously screened. Prevalence of any hrHPV E6/E7 mRNA was 21% (95% confidence interval (CI): 19 to 23%); the prevalence of HPV-16 was 2.6%, HPV-18/45 1.9%, and HPV 16 and 18/45 were jointly found in 0.1% of the study population. Younger age, pregnancy and HIV-positivity were independently associated with any hrHPV infection. Of the 255 evaluable cervical biopsies, the positive predictive value of detecting any hrHPV E6/E7 mRNA for presence of cervical intraepithelial neoplasia grade 2 or higher (“CIN 2+”) was 8.2% (95% CI: 5.1 to 12%). The positive predictive value associated with detection of HPV-16 mRNA (15%) or HPV-18/45 mRNA (15%) was only slightly higher.

**Conclusion:**

Among community-based women in Uganda, the prevalence of any hrHPV E6/E7 mRNA in vaginal samples was high, but the prevalence of the most oncogenic HPV types (16, 18, or 45) was substantially lower. Positive predictive value of hrHPV mRNA-positivity for CIN 2+ was also low, including when restricting to HPV 16/18/45-positivity. The findings emphasize the need to identify more specific screening approaches for cervical cancer.

## Background

Sub-Saharan Africa has the highest rates of cervical cancer worldwide [1], and as the epicenter, the region is in most urgent need for cancer control solutions. Cervical cancer is caused by infection with one or more of 14 high-risk human papillomavirus (hrHPV) types [2], of which three (types 16,18, and 45) contribute to about 77% of all occurrences worldwide [3]. Resource-rich settings have taken advantage of this causal link by incorporating hrHPV DNA testing into cervical cancer screening programs [4]. While highly sensitive for cervical cancer and cervical intraepithelial neoplasia (CIN) (i.e., pre-cancer), hrHPV DNA detection is not specific [5]. Thus, hrHPV-positivity is followed in resource-replete settings by additional testing (e.g., colposcopy) prior to any intervention. In resource-limited settings, hrHPV testing followed directly by treatment, in so-called “screen-and-treat” approaches, is now recommended [2, 6]. The low specificity of hrHPV DNA testing, however, may lead to over-treatment [7], a cost to both the patient [8] and the overstretched health system.

One potential strategy to improve specificity of cervical cancer screening is testing for the mRNA of the oncogenic proteins E6 and E7 from hrHPV types. In data from mainly European and American populations, such hrHPV mRNA tests have demonstrated sensitivity and specificity above 90% for the detection of CIN grade 2 or higher (“CIN 2+”) [9–11]. Detecting HPV 16, 18 or 45 E6/E7 mRNA types enhances specificity even further [12, 13]. Implementation of cervical cancer screening strategies employing hrHPV testing in low-resource countries, especially in sub-Saharan Africa, is in its infancy. Most of the experience to date has used hrHPV DNA tests, and there is limited population-level data [6, 14]. To our knowledge, the only research studying hrHPV mRNA in sub-Saharan Africa found high sensitivity and specificity for high-grade squamous intraepithelial (or more severe) lesions but was conducted in high-risk populations of commercial sex workers and HIV-infected women [15, 16]. How hrHPV mRNA testing performs in broader community-based populations in sub-Saharan Africa is not known.

In order to better understand the utility of hrHPV mRNA testing for population-based screening programs in sub-Saharan Africa, we set forth in Uganda — one of the countries most affected by cervical cancer in the region — to determine a community-based estimate of the prevalence and correlates of hrHPV E6/E7 mRNA detection and the positive predictive value of detection of this biomarker for pre-cancer or cancer.

## Methods

### Overall design

We carried out a cross-sectional study of the prevalence and correlates of hrHPV E6/E7 mRNA in self-collected vaginal specimens among women attending community-based cervical cancer screening fairs in rural western Uganda. Women with detectable hrHPV mRNA subsequently underwent a cervical biopsy to determine presence of CIN or cervical cancer, thus allowing estimation of the positive predictive value of E6/E7 mRNA detection.

### Study population

The target population was women residing in either the Kiboga or Kyankwanzi districts in western Uganda. Both districts are characterized as rural with the largest population center holding 8342 residents. In a randomly chosen 16 communities in these districts, we engaged Village Health Team members (VHTs) to inform and mobilize women to attend cervical cancer screening fairs in central locations in their communities. VHTs are lay citizens that receive basic training by the Ministry of Health and are utilized by the Ministry of Health to promote health programs within their communities. The VHTs made announcements on local radio and at churches, burial ceremonies, markets, and community centers. The mobilization message informed residents about the importance of screening for cervical cancer and the upcoming screening fairs in their communities. On the day of the fair, women attended an interactive educational session about cervical cancer, HPV infection and screening, and all women 25–49 years residing in the two districts were asked to participate in a research study about screening; women with a history of cervical cancer or prior hysterectomy were excluded. The study was approved by institutional review boards in both Uganda and the United States., and all women provided written informed consent to participate.

### Procedures and measurements

#### Socioeconomic, demographic and clinical characteristics

We used an interviewer-administered questionnaire to ask participants about their demographic, socioeconomic, and clinical characteristics, including extensive contact information for result communication and subsequent treatment if necessary.

#### Vaginal specimen collection and handling

Participants were provided with instructions about self-collection of a vaginal specimen, including a graphic illustration. Each woman was given the testing kit (Aptima®, Hologic Inc.) containing a cytobrush and a capped vial containing PreservCyt® medium. They were instructed to insert the cytobrush into their vagina until it met resistance, rotate three times, and then place the brush into the vial and recap. From the field, the specimens were transported at room temperature to Kampala within 1 week and then frozen at -20^o^ C prior to testing.

#### HPV testing

Vaginal samples were tested using the Aptima® HPV assay (Hologic Inc.) at the University of Washington-University of Nairobi STI Laboratory, Mombasa Kenya, based on manufacturer’s instructions. Briefly, the Aptima® assay is a nucleic acid amplification test for the qualitative detection of E6/E7 mRNA from the 14 hrHPV types (16, 18, 31, 33, 35, 39, 45, 51, 52, 56, 58, 59, 66 and 68). Assay results are interpreted on the basis of the signal-to-cut-off ratio for the analyte, and specimens with ratios ≥1.0 were considered positive. All hrHPV-positive samples were further genotyped for HPV-16, HPV-18 and HPV-45 using the Aptima® HPV 16 18/45 genotype assay, also performed according to manufacturer’s instructions.

#### Post-screening clinical follow-up

Women who tested positive for any hrHPV mRNA were asked to return to a mobile treatment unit in their community. Women who presented during their menses were asked to return when their cycle had stopped. After excluding women who were self-reporting or suspicious for pregnancy and confirmed with a positive pregnancy test (MOTI test®, Atlas Link, Beijing, China), we performed Visual Assessment for Treatment (VAT) using 3–5% acetic acid. Women with lesions felt to be inappropriate for cryotherapy or suspicious for cancer were referred for alternative management at Mulago Hospital in Kampala; all others underwent a 2 or 3 mm cervical biopsy (directed or random if normal VAT) before receiving cryotherapy. In other words, in order for us to strive for complete ascertainment of CIN 2+ in all hrHPV RNA-positive women, VAT was only used to determine which women should have a therapy different than cryotherapy, and for these women a biopsy was also sought prior to therapy.

#### Pathologic interpretation

Biopsies (including those obtained from the referred women) were processed at the Department of Pathology of the Makerere College of Health Sciences; they were determined inadequate for histological evaluation if they were too small for processing. Adequate biopsies were interpreted with hematoxylin and eosin staining. Specimens were read as normal, cervicitis, CIN 1, CIN 2/3 or invasive cancer.

### Statistical analysis

After determining the descriptive parameters for the population, we evaluated the participants’ various sociodemographic and clinical characteristics for their association with hrHPV mRNA-positivity (for any of the 14 hrHPV types and for HPV 16, 18, or 45). We used prevalence ratios as the measure of association and used log-binomial regression to estimate the prevalence ratios adjusted for other factors. We used a directed acyclic graph (DAG) to depict background knowledge and inform variable selection in the multivariable regression models [17, 18]. The positive predictive value of mRNA-positivity was defined as the percentage of mRNA-positive vaginal specimens that were CIN 2+ on biopsy. Several definitions of RNA positivity were explored (e.g., any hrHPV-positivity, HPV-16-positivity, etc.). All calculations were performed using Stata version 14.0 (Stata Corp., College Station, Texas).

## Results

### Characteristics of the study population

A total of 2142 women attended and registered for cervical cancer screening at one of 24 health fairs within 16 communities between March and November 2016. Two hundred and forty women were ineligible for the research study due to age (96%), symptoms or exam suggestive of cervical cancer (2.5%), or prior hysterectomy (1.3%). Of the 1902 eligible women, 1892 (99%) provided a self-collected vaginal specimen while 1890 provided both a specimen and a completed questionnaire (Table 1). The median age of the participants was 34 years (interquartile range [IQR] 28–40), most (83%) were married, 79% had no formal or only primary level education, and the vast majority (93%) described their job status as non-professional work (e.g., farming, fishing, or housekeeping). The median number of children was 4 (IQR 3–6), and 66% of women were not currently using a family planning method. Most (90%) participants had undergone HIV testing with 10% self-reporting as HIV-infected, but only 5.0% had ever undergone cervical cancer screening.

**Table 1:** Characteristics of 1892 women from two rural districts of Uganda participating in a community-based study of cervical cancer screening

Characteristic	Percentage
Age, in years	
20–29	32%
30–39	41%
≥ 40	27%
Marital status^a^	
Never married	2.7%
Married	83%
Separated/divorced/widowed	14%
Education^a^	
None	18%
At least some primary	61%
At least some secondary	20%
At least some tertiary	1.0%
Occupation^a^	
Unemployed	79%
Employed, non-professional	14%
Employed, professional	7.4%
Distance of home from screening venue^a^	
< 2 km	60%
3–5 km	29%
> 5 km	11%
Transport to screening venue^b^	
Walked	89%
Other transport	11%
Parity^a^	
0	3.0%
1–3	34%
4–6	40%
> 6	23%
Pregnant^a^	10%
Prior cervical cancer screening^a^	5.0%
HIV-infected, via self-report^c^	9.6%
Using antiretroviral therapy^d^	98%

^a^missing in 2 participants

^b^missing in 19 participants

^c^225 participants reported never testing

^d^among those self-reporting to be HIV-infected

### Prevalence and correlates of hrHPV mRNA-positivity

All the 1892 self-collected vaginal specimens yielded evaluable hrHPV mRNA data. The prevalence of E6/E7 mRNA from any one of the 14 high-risk HPV types was 21% (95% confidence interval (CI): 19 to 23%) (Table 2). Additional reflex testing with the Aptima® 16, 18/45 genotype assay allowed for finer-level delineation of the high-risk types; the prevalence of any of the higher risk genotypes (16, 18 or 45) was 4.6% (95% CI: 3.7 to 5.6%). HPV 16 mRNA prevalence was 2.6% (95% CI: 2.0 to 3.5%), HPV 18/45 was 1.9% (95% CI: 1.3 to 2.6%) (Table 2) and two participants 0.11% (95% CI: 0.01 to 0.38%) tested positive for both hrHPV 16 and 18/45. The collective prevalence of the other hrHPV types (31, 33, 35, 39, 51, 52, 56, 58, 59, 66 and 68) without 16, 18 or 45 was 16% (95% CI: 14 to 18%) (Table 2).

**Table 2:** hrHPV prevalence, by self-reported HIV infection status, among women from two rural districts of Uganda participating in a community-based study of cervical cancer screening

hrHPV Type Detected	HIV-infected^c^ Participants (*N* = 160) % (95% CI)	HIV-uninfected Participants (*N* = 1507) % (95% CI)	HIV-untested Participants (*N* = 225) % (95% CI)	All Participants (*N* = 1892) % (95% CI)
Any hrHPV	40% (32 to 48%)	19% (17 to 21%)	17% (12 to 22%)	21% (19 to 23%)
At least one of HPV-16, 18 and 45^a^	11% (6.3 to 16%)	4.0% (3.1 to 5.2%)	4.0% (1.8 to 7.5%)	4.6% (3.7 to 5.6%)
HPV-16^a, b^	4.4% (1.8 to 8.8%)	2.7% (1.9 to 3.6%)	2.2% (0.7 to 5.1%)	2.7% (2.1 to 3.6%)
HPV-18 and/or 45^a, b^	6.9% (3.5 to 12%)	1.5% (0.9 to 2.2%	1.8% (0.5 to 4.5%)	1.8% (1.3 to 2.6%)
At least one of HPV-31, 33, 35, 39, 51, 52, 56, 58, 59, 66 and 68 but without 16, 18 and 45	29% (22 to 36%)	15% (13 to 17%)	13% (8.8 to 18%)	16% (14 to 18%)

^a^may also include HPV-31, 33, 35, 39, 51, 52, 56, 58, 59, 66 and 68

^b^Includes 2 patients who were positive for HPV-16 and HPV-18/45

^c^HIV infection status ascertained by self-report

**Fig. 1:**
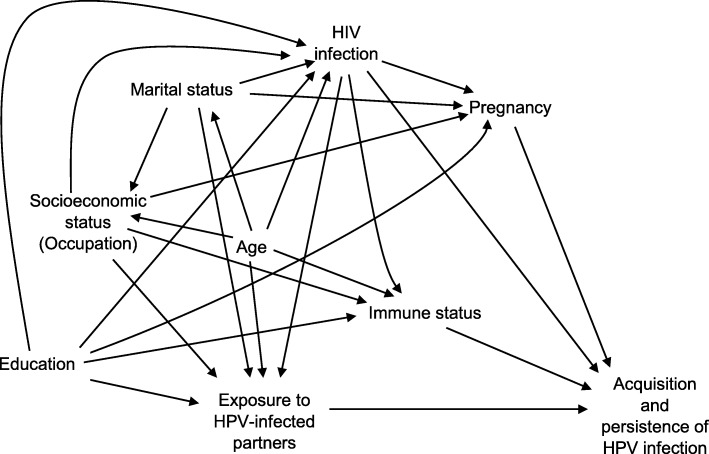
A directed acyclic graph (DAG) depicting our hypothesized conception of the system under investigation. We sought to estimate the independent contribution of age, marital status, education, occupation (proxy for socioeconomic status), pregnancy, and HIV Infection status to the prevalence of HPV infection in Ugandan women. Exposure to HPV-infected sexual partners and immune status were not directly measured by our study and hence could not be evaluated. The DAG was used to guide which variables to control for when assessing the independent contribution of the various constructs

In evaluating independent correlates of the presence of E6/E7 HPV mRNA, we constructed a directed acyclic graph to depict the system and inform what factors to adjust for (Fig. 1). We first evaluated any hrHPV type mRNA-positivity, and we found, in unadjusted analyses, that younger age, secondary level education, professional employment, pregnancy state and HIV infection were all associated with mRNA-positivity (Table 3). HIV infection had the largest magnitude of association (unadjusted prevalence ratio (PR) =2.07, 95% CI 1.67 to 2.57). After relevant multivariable adjustment, we found that younger age, pregnancy and HIV infection remained significantly associated with hrHPV mRNA-positivity. Each added year of age was associated with a 3.0% decreased probability of testing hrHPV-positive (95% CI 1.0 to 4.0%, *P* < 0.001). Women who were pregnant were 1.37 times as likely to be hrHPV-positive than women who were not pregnant (95% CI 1.04 to 1.80, *P* = 0.02). HIV-infected participants were over two times more likely to be hrHPV-positive than those who were HIV-uninfected (PR 2.20; 95% CI 1.74 to 2.78; *P* = < 0.001). When evaluating HPV 16, 18, or 45 mRNA-positivity, we found, in adjusted analyses, that only younger age and HIV infection status were significantly related to hrHPV mRNA detection.

**Table 3:** Evaluation of potential independent correlates of hrHPV E6/E7 mRNA-positivity among Ugandan women participating in a community-based study of cervical cancer screening. Separate analyses are shown for correlates of any hrHPV type and for a restricted set of HPV 16 or 18/45

Characteristic	Any hrHPV Type Prevalence Ratio (95% CI)				HPV 16 or 18/45 Prevalence Ratio (95% CI)			
	Unadjusted	*P* value	Adjusted	*P* value	Unadjusted	*P* value	Adjusted	*P* value
Age, per additional year^a^	0.97 (0.96 to 0.99)	< 0.001	0.97 (0.96 to 0.99)	< 0.001	1.20 (0.92 to 1.58)	0.18	0.96 (0.92 to 0.99)	0.024
Marital status^b^								
Never married	Ref.		Ref.		Ref.		Ref.	
Married	0.71 (0.45 to 1.13)	0.15	0.87 (0.50 to 1.52)	0.50	1.15 (0.28 to 4.55)	0.84	1.19 (0.30 to 4.74)	0.81
Separated/divorced/widowed	0.96 (0.59 to 1.57)	0.17	1.02 (0.56 to 1.85)	0.95	1.36 (0.32 to 5.81)	0.68	1.30 (0.29 to 5.76)	0.73
Educational level^c^								
None	Ref.		Ref.		Ref.		Ref.	
As least some primary	1.28 (0.97 to 1.69)	0.079	1.17 (0.86 to 1.58)	0.32	0.94 (0.51 to 1.73)	0.84	0.73 (0.38 to 1.38)	0.33
At least some secondary	1.39 (1.01 to 1.91)	0.041	1.12 (0.78 to 1.63)	0.52	1.58 (0.82 to 3.08)	0.17	0.99 (0.46 to 2.14)	0.98
At least some tertiary	1.68 (0.77 to 3.69)	0.19	1.36 (0.58 to 3.17)	0.48	2.64 (0.64 to 10.4)	0.17	1.69 (0.35 to 8.10)	0.51
Occupation^d^								
Unemployed	Ref:		Ref:		Ref.		Ref.	
Non-professional	1.17 (0.89 to 1.56)	0.26	1.08 (0.79 to 1.46)	0.63	1.64 (0.90 to 2.99)	0.11	1.35 (0.68 to 2.67)	0.39
Professional	1.44 (1.12 to 1.84)	0.004	1.33 (0.98 to 1.80)	0.068	2.13 (1.25 to 3.63)	0.005	1.56 (0.77 to 3.15)	0.21
Pregnancy status^e^								
Not pregnant	Ref.		Ref.		Ref.		Ref.	
Pregnant	1.31 (1.02 to 1.69)	0.036	1.37 (1.04 to 1.80)	0.026	1.14 (0.60 to 2.17)	0.69	1.30 (0.67 to 2.52)	0.43
HIV infection status^f^								
HIV-uninfected	Ref.		Ref.		Ref.		Ref.	
HIV-infected	2.07 (1.67 to 2.57)	< 0.001	2.20 (1.74 to 2.78)	< 0.001	2.62 (1.57 to 4.38)	< 0.001	3.08 (1.77 to 5.35)	< 0.001

^a^Age was adjusted for education, HIV infection status, marital status, pregnancy, and occupation

^b^Marital status was adjusted for age, education, HIV infection status, pregnancy, and occupation

^c^Education was adjusted for age, HIV infection status, marital status, pregnancy, and occupation

^d^Occupation was adjusted for age, education, HIV infection status, marital status, and pregnancy

^e^Pregnancy was adjusted for age, education, HIV infection status, marital status and occupation

^f^HIV infection status was adjusted for age, education, marital status, pregnancy, and occupation

### Positive predictive value of HPV E6/E7 mRNA-positivity for CIN 2+

A total of 301 of 393 (77%) hrHPV mRNA-positive participants returned for treatment, of whom 38 did not receive a cervical biopsy or cryotherapy because of pregnancy (71%), vaginal prolapse (5.7%), inability to safely administer cryotherapy due to cervical position (11%), referral to Mulago Hospital for alternative management but subsequent loss to follow-up (11%), or deferral because of concurrent systemic illness (2.7%). There was no significant difference in age, marital status, educational level, parity and HIV status between those women who returned and those who did not (data not shown). Of the 263 participants who underwent a biopsy, 255 biopsy samples (including 7 performed at the referral hospital) were adequate for interpretation. Among these, 8.2% (95% CI: 5.1 to 12%) displayed CIN 2+ (4.7% CIN 2, 2.7% CIN 3 and invasive cancer 0.8%), making the positive predictive value of detecting any hrHPV E6/E7 mRNA for the presence of CIN 2+ 8.4% (Table 4). The estimates for the positive predictive values associated with detection of HPV-16 and/or HPV-18/45 E6/E7 mRNA were only slightly higher but, because of smaller sample size, considerably less precise.

**Table 4:** Positive predictive value, by self-reported HIV infection status, of detecting hrHPV E6/E7 mRNA for the presence of CIN 2+ among women from two rural districts of Uganda participating in a community-based study of cervical cancer screening

hrHPV Type Detected	HIV-infected^b^ Participants			HIV-uninfected Participants			HIV-untested Participants			All Participants		
	Total No.	No. with CIN 2+	Positive predictive value % (95% CI)	Total No.	No. with CIN 2+	Positive predictive value % (95% CI)	Total No.	No. with CIN 2+	Positive predictive value % (95% CI)	Total No.	No. with CIN 2+	Positive predictive value% (95% CI)
Any hrHPV	42	4	9.8% (2.7 to 23%)	193	12	6.3% (3.3 to 11%)	20	5	25% (8.7 to 49%)	255	21	8.2% (5.1 to 12%)
At least one of HPV-16, 18 and 45^a^	10	1	10% (0.25 to 45%)	43	4	9.5% (2.7 to 23%)	5	4	80% (28 to 99%)	58	9	15% (7.3 to 27%)
HPV-16^a^	4	0	0% (0 to 60%)	28	3	11% (2.3 to 28%)	2	2	100% (16 to 100%)	34	5	15% (5.0 to 31%)
HPV-18 and/or 45^a^	7	1	14% (0.36 to 58%)	16	1	6.3% (0.15 to 30%)	3	2	67% (9.4 to 99%)	26	4	15% (4.4 to 35%)
At least one of HPV-31, 33, 35, 39, 51, 52, 56, 58, 59, 66 and 68 but without 16, 18 and 45	32	3	9.1% (1.9 to 24%)	150	8	29% (22 to 37%)	15	1	13% (1.7 to 40%)	197	12	6.1% (3.2 to 10%)

^a^may also include HPV-31, 33, 35, 39, 51, 52, 56, 58, 59, 66 and 68

^b^HIV infection status ascertained by self-report

## Discussion

In sub-Saharan Africa, screening for cervical cancer with hrHPV testing is relatively new and most strategies have utilized hrHPV DNA assays. We evaluated the prevalence, positive predictive value and correlates of hrHPV E6/E7 mRNA in community-based screening in rural Uganda. We found that the prevalence of any hrHPV E6/E7 mRNA was substantial (21%), but there was a much lower prevalence (4.6%) of HPV-16 or 18/45, the types traditionally recognized as most oncogenic. The positive predictive value of hrHPV E6/E7-positiivity for CIN 2+ was also low (8.2%) without much improvement when limited to HPV-16 or HPV-18/45 E6/E7 mRNA-positivity. Younger age, pregnancy and HIV-positivity were significantly associated with harbouring any hrHPV mRNA.

The prevalence of hrHPV mRNA in our study was substantially lower than what was found by other hrHPV mRNA studies within Africa. In a study by Adamson et al. among 325 HIV-infected women in South Africa, the prevalence of hrHPV mRNA was 37% [15]. Ting et al. found a 30% prevalence of hrHPV mRNA among 344 female sex workers in Kenya [16]. The high-risk nature of these populations likely explains their higher prevalence. Our hrHPV mRNA prevalence is similar to what has been documented for the prevalence of hrHPV DNA (17–19%) [19] in four other rural population-based studies in Uganda. Given that HPV DNA detection is typically greater than HPV mRNA detection within various populations studied [20–24], this may mean that our population has a higher burden of hrHPV infection than previously recognized in rural Uganda. This may be explained by intrinsically higher endemicity of hrHPV or differences in the distributions of key causal determinants such as age or HIV infection. Alternatively, the burden of hrHPV infection may be equivalent in our population and others studied in the past, but some aspect of the population is causing hrHPV mRNA to be more commonly expressed in our participants (thus closing the gap between hrHPV mRNA and DNA detection). If this latter explanation is operative, it could, in part, explain the low positive predictive value for CIN 2+ that we observed. Finally, our finding of the prevalence for HPV-16 (2.6%) did not substantively differ from what has been documented in other rural population-based studies within Uganda (HPV-16 DNA 2.1% [25] and 4.5% [26]). We are not aware of other population-based estimates of HPV 18 or 45 prevalence in rural Uganda.

The positive predictive value of the presence of any hrHPV mRNA for CIN 2+ that we observed was low but very similar to what was observed by Ting et al. in Kenya (10%) [16] and within a screening population in the United Kingdom (6.3%) [9]. However, our positive predictive value was substantially lower than that found for hrHPV mRNA-based testing among women aged 20–65 years who attended private screening clinics in Paris (19%) [23] or among population-based women aged 25–65 years in British Columbia, Canada (16%) [21]. Our estimate was also, surprisingly, not higher than studies using hrHPV DNA testing in other population-based studies in Africa, including Uganda (11% among women mean age 37 years [6]), Rwandan (12% among women aged 25–69 years [27]), Cameroon (10% among women aged 30–65 years [28]), and Zimbabwean (19% among women aged 25–55 years [29]). Compared to all these latter studies (mRNA and DNA HPV-based), we cannot decipher whether the differences in the positive predictive values are because of differences in prevalence of the disease state (CIN 2+), differences in the prevalence of hrHPV E6/E7 mRNA-positivity without CIN, or differences in the inherent sensitivity or specificity of the assays for CIN 2+ [24]. Nonetheless, all of these positive predictive value estimates are low in an absolute sense, undoubtedly would result in needless treatment of many women if reflexively used in screen-and-treat strategies, and bespeak of the need for more specific biomarkers. This is especially the case in settings that can afford frequent serial screening (e.g., every 2 to 5 years). In settings where frequent screening, however, is not feasible, these positive predictive values need to be interpreted differently. That is, a woman’s lifetime risk of cervical cancer given infection with hrHPV needs to be considered, not just the concurrent presence of CIN 2+. Indeed, HPV screening and reflexive cryotherapy was found to be a more effective screening strategy than both VIA and pap screening in reducing cervical disease and deaths from cancer even when done once in a lifetime [30–32]. Therefore, in resource-limited settings where screening may happen only once in a lifetime if at all, the potential benefits of HPV-based screen-and-treat may trump concerns of overtreatment.

We found that younger age, pregnancy and HIV-positivity were significantly associated with detecting hrHPV mRNA. The association with HIV is consistent with multiple reports in which HIV infection results in increased incidence of HPV infection [33, 34], decreased ability to clear existing infections [34, 35], and possibly reactivation of latent HPV infection [36]. All of these data reinforce the need to prioritise cervical cancer control among HIV-infected populations. That pregnancy was associated with hrHPV-positivity may simply be related to recent and/or increased levels of sexual activity, but there are pregnancy-related endocrine factors which may affect acquisition and clearance of hrHPV, and there is potential for increased HPV production and shedding in the setting of the increased ectropion seen in pregnancy [37, 38]. Yet, not all studies have found this association [39]. Furthermore, because pregnant women are not candidates for screen-and-treat strategies, the practical implications of pregnancy as a determinant for HPV-positivity are minimal, and there are no studies, to our knowledge, showing an increased risk for CIN 2+ in pregnancy. Finally, our finding that hrHPV-positivity was reduced with older age is consistent with other studies using hrHPV mRNA [40] and DNA [41] although they were among women with normal cytology. Despite the decline in HPV prevalence with age, the risk of HPV-related disease (pre-cancer and invasive cancer) increases with age, which forms the basis of the current 2013 WHO guidelines suggesting that HPV screening start at 30 years of age [42].

Our approach sought to estimate the prevalence, correlates and predictive value of hrHPV mRNA at the community level, but there were limitations. First, our participants freely responded to a community mobilization campaign, which may have selectively enriched the study population for those who perceived themselves to be at risk of HPV infection or may be more likely to engage in prevention behaviours. It is therefore difficult to assess whether the participants were strictly representative of the general Ugandan rural population. Second, we could not with our study design estimate the negative predictive value of non-detection of hrHPV mRNA. Doing so would have required biopsy of all or a representative sample of the mRNA-negative women, which was beyond the scope of our work. Third, because of the nature of our recruitment and screening (large numbers of participants screened simultaneously), we were not able to comprehensively measure some of the constructs (e.g., socioeconomic status) potentially related to hrHPV-positivity. Thus, our lack of finding a role for some of the constructs may have been because of misclassification. Finally, the cross-sectional nature of the study precluded a better understanding of the meaning of our correlations (i.e., were they influencing HPV acquisition and/or persistence) or of the positive predictive value of mRNA-positivity for CIN 2+ over a longer duration.

## Conclusion

In this community-based sample of women from Uganda, the prevalence of any hrHPV E6/E7 mRNA was high, but the prevalence of the most oncogenic HPV types (16, 18, or 45) was substantially lower. Positive predictive value of hrHPV mRNA-positivity for CIN2+ was also low, including when limited to just HPV 16/18/45-positivity. The findings emphasize the need to identify more specific screening approaches for the prevention and early detection of cervical cancer.

## Abbreviations

CI: Confidence interval
CIN: Cervical intraepithelial neoplasia
DAG: Directed acyclic graph
DNA: Deoxyribonucleic acid
E6: Early protein 6
E7: Early protein 7
HIV: Human immunodeficiency virus
HPV: Human papillomavirus
hrHPV: High-risk human papillomavirus
IQR: Interquartile range
mRNA: Messenger ribonucleic acid
UNCST: Uganda National Council for Science and Technology
VAT: Visual assessment for treatment
VHTs: Village Health Team members
WHO: World Health Organisation
